# Combined Argon Laser and Low Dose Acetylsalicylic acid in Treatment of Acute Central Serous Chorioretinopathy

**Published:** 2018

**Authors:** Alahmady Hamad Alsmman, Engy Mohamed Mostafa, Amr Mounir

**Affiliations:** 1 Sohag Faculty of Medicine, Ophthalmology Department, Sohag, Egypt

**Keywords:** Central Serous Chorioretinopathy, Argon Laser, Acetylsalicylic Acid, Macular Thickness, Optical Coherence Tomography (OCT)

## Abstract

This study was designed to evaluate the efficacy of low-dose of oral acetylsalicylic acid (aspirin) with focal argon laser for the treatment of acute central serous chorioretinopathy (CSCR). In this prospective case-control study, 40 Patients with acute CSCR were classified randomly to two groups; group A with no treatment as the control group and group B with argon Laser in focal treatment once, followed by aspirin, 100 mg per day orally, with follow up period of 12 months by evaluation of visual acuity, and by Optical Coherence Tomography (OCT), every three months for one year. Patients in the second group treated with argon Laser and aspirin showed more clinically significant improvement in both visual acuity and OCT macular thickness by the end of the follow-up period when compared with the observational group. It was concluded that argon Laser with low-dose oral aspirin results in improvement of visual acuity and OCT macular thickness.

## INTRODUCTION

Central Serous Chorioretinopathy (CSCR) is a posterior segment disease that usually effects young males in their third and fourth decade of life [1, 2]. Neurotic patients, or those, who are experiencing psychological stress were at higher risk of developing CSCR [3, 4]. Fluid accumulation between the neuroretina and Retinal Pigment Epithelium (RPE) is the main pathophysiology of CSCR. Its predilection of site is the posterior pole, leading to early central vision loss, scotoma, metamorphopsia, and/or micropsia [5, 6].

Fluorescein Angiography (FA) is of great value in the diagnosis of CSCR, confirming the focal point of leakage with fluorescein diffusion in the form of a ‘smokestack' pattern, or ‘expansile dot pattern ' appearance under a serous neuroretinal detachment. The CSCR may be solitary or have multiple leaking points [7-9]. Optical Coherence Tomography (OCT) is helpful in visualization of morphologic changes occurring in the retinal layers and the detachment occurring in the RPE, while it could also be used to track the disease evolution [10-13].

 Observation is the first line treatment of CSCR, as the disease is self-limiting with non-permanent effects of the first episodes of acute CSCR being likely in most patients. The patient should be followed-up regularly to confirm resolving of the neurosensory detachment, which normally occurs within a period of three to four months, however, the rate of recurrence is considerably high [5, 14].

Focal argon laser photocoagulation application to the point of leakage has become a vastly practiced line of management for acute CSCR. The treatment aims to produce a ‘sealing' of the RPE defect, guided by fluorescein angiography. Furthermore, the retinal laser photocoagulation stimulates closer RPE cells, thus, promotes the pump function of the RPE [15-17].

Acetylsalicylic acid (Aspirin), at a low dose, has been reported to be beneficial in the treatment of CSCR through its anti-platelets effects and by reducing serum levels of Plasminogen Activator Inhibitor 1 (PAI-1) [18, 19]. The aspirin treatment effect depends on the hypothesis of impaired fibrinolysis in conjunction with augmented platelet aggregation in the choriocapillaris in CSCR pathogenesis [20].

To the best of the author’s knowledge, there are no studies, which have evaluated the effectiveness of combined focal argon laser with low-dose of oral aspirin in treatment of Central Serous Chorioretinopathy (CSCR). The purpose of this prospective study was to evaluate the efficacy of both laser and aspirin together, in comparison to observation alone.

## MATERIALS AND METHODS

This was a prospective case-control study, including patients with acute CSCR, presented to the Ophthalmology Department of Sohag University Hospital, Egypt, between January 2016 and July 2016. Exclusion criteria were presence of ocular or retinal disease other than CSCR, history of previous attacks of CSCR, history of coagulation problems or bleeding tendencies, previous retinal laser photocoagulation, history of peptic ulcers, pregnancy, or aspirin allergy.

Forty eyes of 40 patients with CSCR were classified randomly to two groups, according to their involved eye being right or left. Patients with pathology in their right eye were assigned to group A and when CSCR was in the left eye, patients were assigned to group B. Each group included twenty patients; group A with no treatment as the control group and group B with argon laser as the focal treatment once, followed by aspirin 100 mg per day, orally, for 12 months.

The Declaration of Helsinki was respected throughout the study. Approval was obtained from Sohag Faculty of Medicine committee and all patients signed written informed consents for the treatment sequence.

 All patients were subjected to complete ophthalmic evaluation, including Corrected Distance Visual Acuity (CDVA) measurement by the Snellen chart, which was converted to a logarithm of the minimum angle of resolution (logMAR), measurement of the Intraocular Pressure (IOP), using I Care tonometer, indirect ophthalmoscopy (Heine 2000 Ophthalmoskop, Heine; 30-D lens, Zeiss), slit lamp biomicroscopy, FA (TRX fundus camera, Topcon Medical Systems, Inc.), and macular OCT (OPTOVUE RTvue) at the first-time visit (baseline).

Fluorescein angiography was used to identify the leakage point and Volk area centralis contact lens (Keeler Ltd, Clewer Hill Road, Windsor, Berks, SL4 4AA) laser was applied using spot size of 100 microns, duration of 100 to 200 milliseconds, and power of 150 to 400 milli Watts (mW) in all subjects.

Follow-up of all patients was done over a period of twelve months; follow up visits were scheduled on the first, third, sixth, and twelfth month. The follow up regimen included CDVA in logMAR, IOP, slit lamp biomicroscopy, indirect ophthalmoscopy, fundus photography, and Central Macular Thickness (CMT) by macular OCT, done during every visit.

Statistical analysis was conducted by SPSS for Windows version 10.0 software (SPSS Inc., Chicago, IL, USA) to compare parameters of both groups and a P value of ≤0.05 was considered statistically significant.

## RESULTS

A total of 40 eyes of 40 patients were enrolled for this prospective study, 29 were males (72.5%) and 11 females (27.5%). The mean ± standard deviation (SD) age was 29.8 ± 4.8 years (range 19 to 40). Demographic and initial visit data for both groups are shown in Table 1, which were insignificant.

**Table 1:** **Demographic and Baseline Visit Data for Both Groups**

	Group A	Group B	P Value
Eyes (n)	20	20	
Age, years (mean ± SD)	26.3 ± 3.7	29.4 ± 4.9	0.731
Gender			
** Male (n)**	13	16	
** Female (n)**	7	4	
Mean CDVA (Log MAR) (mean ± SD)	0.532 ± 0.16	0.541 ± 0.136	0.894
Mean CMT in microns	468.1 ± 28.32	453.9 ± 34.65	0.764

Data are presented as Mean ± SD M: male; F: female; Log MAR: logarithm of the minimum angle of resolution; CDVA: corrected distance visual acuity; CMT: central macular thickness; SD: standard deviation; n: number

**Table 2:** **Mean Corrected Distance Visual Acuity** **Follow up Data for Both Groups in One Year**

	Group A	Group B	P Value
Pre mean Log MAR CDVA	0.532 ± 0.16	0.541 ± 0.136	0.894
Post 1m mean Log MAR CDVA	0.478 ± 0.12	0.309 ± 0.102	0.187
Post 3m mean Log MAR CDVA	0.412 ± 0.141	0.246 ± 0.139	0.081
Post 6m mean Log MAR CDVA	0.387 ± 0.11	0.211 ± 0.121	0.064
Post 12m mean Log MAR CDVA	0.373 ± 0.21	0.174 ± 0.181	** 0.034** *

Data are presented as Mean ± SD

SD: standard deviation; CDVA: corrected distance visual acuity; Log MAR: logarithm of the minimum angle of resolution; m: months.

* P values less than 0.05

**Table 3:** **Mean Central Macular Thickness (CMT) Follow up Data for both Groups During One Year**

	Group A	Group B	P Value
Pre mean CMT	468.1± 28.32	453.9±34.65	0.764
Post 1m mean CMT	332.1± 26.32	311.9±30.25	0.257
Post 3m mean CMT	305.1± 28.32	283.9±30.65	0.066
Post 6m mean CMT	301.1± 34.32	276.9±27.61	** 0.049** *
Post 12m mean CMT	281.1± 29.12	262.2±31.75	** 0.034** *

Data are presented as Mean ± SD

SD: standard deviation; CMT: central macular thickness; m: months.

* P values less than 0.05

**Figure 1:**
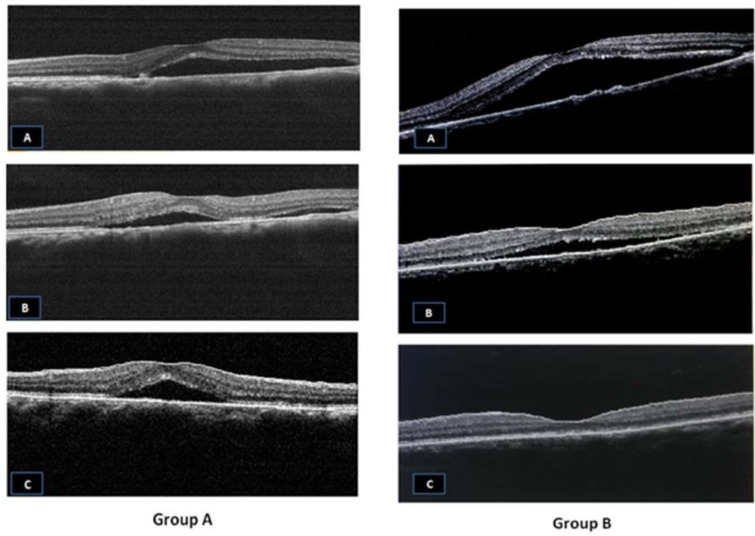
Group A: A) At diagnosis: Macular detachment due to acute Central serous chorioretinopathy (CSCR). B,C) At 6th and 12th month, respectively: Minimal improvement with no resolution. Group B: A) At diagnosis: Macular detachment due to acute CSCR. B,C) At 6th and 12th months, respectively: Marked improvement with complete resolution.

**Figure 2:**
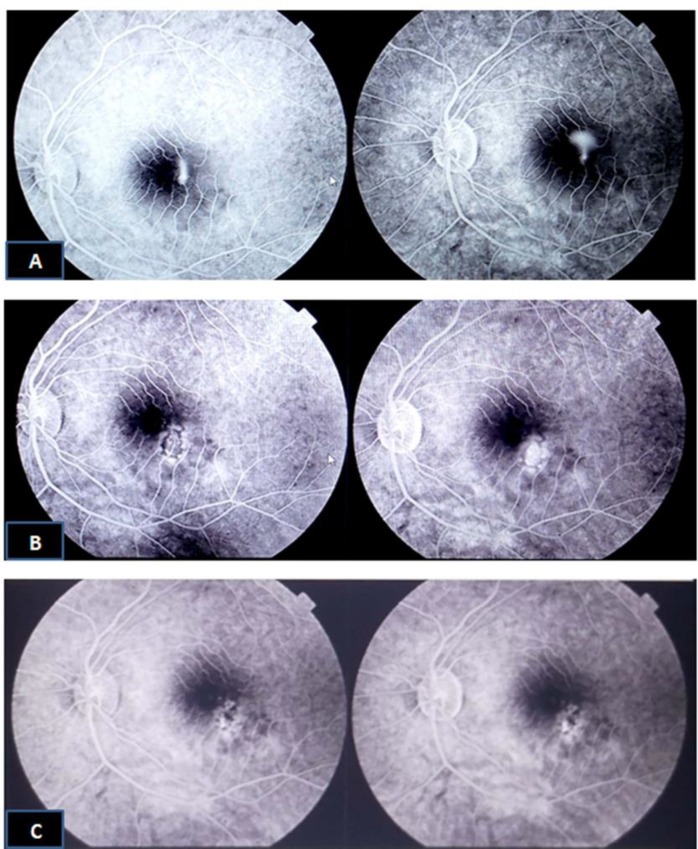
Fluorescein Angiography of Acute Central Serous Chorioretinopathy (CSCR). A) At diagnosis: Macular Leakage due to acute CSCR. B,C) At 6th and 12th months, respectively: Marked improvement with no leakage after combined argon laser and low dose aspirin treatment.

The mean CDVA follow up data for one year, as summarized in Table 2, improved in both groups with no statistically significant difference in all follow ups, except at 12 months, when there was a statistically significant difference between both groups with greater improvement in group B (P=0.034).

The mean CMT follow up data for one year, as summarized in Table 3, improved in both groups with statistically significant difference only at the six (P=0.049) and twelve-month (P=0.034) follow up with more improvement in group B (Figures 1 and 2).

Four cases showed persistence of CSCR with no improvement and two cases of recurrence were identified in group A. Group B showed no cases of persistence of the CSCR and one case of recurrence (Table 4). 

**Table 4:** **Number of recurrences and persistent central serous chorioretinopathy** **cases in both groups**

	Group B	Group A
Persistence of the CSCR	4	0
Recurrence of the CSCR	2	1

CSCR: Central serous chorioretinopathy.

## DISCUSSION

In Central Serous Chorioretinopathy (CSCR) serous detachment of the neurosensory retina occurs over an area of choriocapillary leakage, through the RPE. The pathophysiology is not well understood, however, focal choroidal vasculopathy and hyperpermeability areas are proposed hypotheses of focal choroidal vascular compromise. Some researchers stated that initial choroidal vascular compromise leads to secondary dysfunction of the overlying RPE [19, 21, 22].

The focus of attention in the current study was the effect of combination of both surgical and medical treatment in cases of classic CSCR. The surgical intervention was focal argon laser therapy by FA guide, and medical treatment was long-term low dose of aspirin. This study attempted to evaluate the effectiveness of this new technique by studying the level of improvement either clinically by CDVA or anatomically by measurement of CMT by OCT.

For a long time, argon laser photocoagulation alone has been an effective method for management of CSCR. Many studies confirmed the efficacy and safety of focal laser photocoagulation to the leaking RPE by FA guide [23]. The current researchers agreed with Novak et al. in usage of argon laser for photocoagulation [24], however, many alternatives for photocoagulation were used: Mitsui et al. [25] used Xenon laser while Slusher et al. [26] used Krypton Laser, Elsner et al. used photodynamic therapy (PDT) [27], and GuptaB et al. used micropulse diode laser [28]. Transpupillary Thermotherapy (TTT) by a 810-nm long-pulse low-energy diode laser was also used. The mechanism of action was increasing the temperature of the choroid and outer retina and sparing the vital inner retina and photoreceptors to some extent, however, the exact mechanism is not obvious [29, 30].

In the current study, the researchers demonstrated improvement in both duration and final outcome, however, Fok et al. [31] reported that laser photocoagulation hastened the resolution, yet had no effect on the final visual outcome nor the rate of recurrence; this discrepancy may be due to addition of aspirin in the current case group. 

This study agrees with Spalter’s study, which reported effectiveness of the Argon laser photocoagulation for acute CSCR in clearly defined focal leakage point on FA. The regenerating cells then slide over and obliterate the pigment epithelial defect responsible for the lesion. However, in his study, side effects, such as laser scar formation, permanent scotoma, and laser-induced choroidal neovascularization was reported, which was not recorded in the current study; this may be due to low energy used with minimal power shots [32].

In the current study, the researchers used medical treatment in the form of aspirin, orally, at a dose of 100 mg per day for 12 months. Caccavale et al. [18] used aspirin, orally, at the same dose for six months and reported that treatment with low-dose aspirin may result in speeding of visual rehabilitation and decreasing incidence of recurrence in CSCR cases compared with the untreated control group, which was parallel with the current findings.

In the current study, the mean CDVA in group B (Aspirin and laser) at diagnosis was 0.541 ± 0.136, which improved to 0.174 ± 0.181 at 12 months (p value = 0.034). This result coincides with a previous study, which suggested that effectiveness of aspirin therapy impaired fibrinolysis and increased platelet aggregation hypothesis in the choriocapillaris, as the possible cause of the pathogenesis of CSCR [20]. Kurup et al. used low-dose of methotrexate [23] and Forooghian et al. [33] used finasteride mineralocorticoid receptor antagonist, yet both studies did not achieve encouraging results. Lim et al. [34] found no positive effect for intraocular bevacizumab injection in classic CSCR cases compared with the control group.

Meyerle et al. [35] also used ketoconazole as a medical treatment for CSCR to decrease endogenous cortisol synthesis, however, they noted that there is no improvement in outcome in a preliminary study or in their later clinical trial.

Also, the significant improvement of mean CDVA in group B coincides with the study of Burumcek et al., [8] in which the final Best-Corrected Visual Acuity (BCVA) was better (P value = 0.003) in the CSCR group treated with laser photocoagulation than that of the control group. Regarding changes in the CMT, in group B, mean CMT decreased by 453.9 ± 34.65 microns from diagnosis to the 12th month with statistically significant differences in the 6th and 12th month follow up, in comparison to Group A (P value = 0.049; P value = 0.034, respectively).

On the whole, it was noted that the functional improvement (represented in the CDVA) and anatomical improvement (OCT macular thickness) in group B treated by both argon laser and aspirin were more than group A. The main limitation of the current study was the relatively small number of cases and short period of follow up. Further studies are warranted with larger sample sizes and longer follow up periods. More groups with different treatment modalities can be established to evaluate the efficacy of each treatment line.

## CONCLUSION

Argon laser combined with low-dose oral aspirin resulted in greater improvement both clinically, by visual acuity, and anatomically, by OCT macular thickness measurement, compared to observation alone, however, future randomized clinical trials with a larger sample size and longer follow up period are recommended.
